# Pilose Antler Protein Extract Alleviates Osteoporosis and Is Associated with Activation of the Wnt/β-Catenin Signaling Pathway

**DOI:** 10.3390/ph19050665

**Published:** 2026-04-24

**Authors:** Junxiao Gong, Yuman Ma, Jun Huang, Wenyu Yang, Yi Wang, Yanan Sun

**Affiliations:** Experimental Research Center, China Academy of Chinese Medical Science, Beijing 100700, China; gongjunxiao2000@163.com (J.G.); yuman322696@163.com (Y.M.); huangjuneve@163.com (J.H.); yangwenyu202502@163.com (W.Y.)

**Keywords:** pilose antler protein extract, osteoporosis, osteoblast precursor cells, osteogenic differentiation, Wnt/β-Catenin pathway

## Abstract

**Objectives**: Pilose antler protein extract (PAE) was investigated for its therapeutic efficacy against ovariectomy (OVX)-induced osteoporosis and its underlying molecular mechanism. **Methods**: An OVX rat model was established to evaluate the effects of PAE on bone microarchitecture, histopathological changes, and bone metabolism-related parameters. Bone structure was assessed using Micro-CT and histological analysis, and biochemical and bone turnover markers were quantified. In vitro, Mouse calvarial pre-osteoblast subclone 14 (MC3T3-E1) Subclone 14 cells were used to examine the effects of PAE on cell viability, proliferation, osteogenic differentiation, and osteogenesis-related protein expression. **Results**: High-dose PAE markedly improved trabecular bone microarchitecture in OVX rats, as reflected by increased bone surface area and bone volume fraction and reduced trabecular separation. PAE significantly enhanced bone calcium content and elevated serum Bone Morphogenetic Protein 2 (BMP-2) and Procollagen Type I N-Terminal Propeptide (PINP) levels, while decreasing serum Alkaline Phosphatase (ALP) activity and C-Terminal Telopeptide of Type I Collagen (CTX-I) levels, indicating a shift toward bone formation. Mechanistically, PAE activated the Wnt-3a/β-Catenin signaling pathway in bone tissue and MC3T3-E1 cells, as evidenced by increased expression of Wnt-3a and β-Catenin proteins. In vitro experiments further demonstrated that PAE promoted MC3T3-E1 cell proliferation and upregulated osteogenic markers, i.e., Runt-related transcription factor 2 (RUNX2) and Osteocalcin (OCN). **Conclusions**: Collectively, these findings suggest that PAE exerts pronounced anti-osteoporotic effects and is associated with activation of the Wnt/β-Catenin signaling pathway.

## 1. Introduction

Osteoporosis (OP) is a systemic disease characterized by reduced bone mass and deterioration of bone microarchitecture, and it is particularly prevalent among postmenopausal women. The disease markedly increases fracture risk, thereby imposing a substantial burden on patients’ quality of life and healthcare systems worldwide. According to The Lancet Diabetes & Endocrinology, more than 8.9 million osteoporotic fractures occur annually across the globe, making osteoporosis prevention and treatment a major public health concern [1,2]. Current clinical therapies primarily focus on inhibiting bone resorption, including bisphosphonates and Receptor Activator of Nuclear Factor Kappa-B Ligand (RANKL) inhibitors, whereas agents that directly stimulate bone formation remain relatively limited. Therefore, the development of safe and effective natural compounds capable of promoting bone formation and restoring bone homeostasis has become an important research focus.

Pilose antler, a traditional Chinese medicinal material used for “kidney tonification and bone strengthening,” possesses unique regenerative capacity and is rich in proteins, peptides, and various growth factors. Increasing pharmacological evidence suggests that pilose antler and its extracts exhibit significant potential in promoting osteoblast proliferation and differentiation and in improving bone metabolism [3,4,5]. Osteogenesis and osteoblastic differentiation are crucial physiological processes for maintaining bone homeostasis, repairing skeletal damage, and preventing degenerative diseases such as osteoporosis.

The Wnt/β-Catenin signaling pathway plays a central role in regulating osteoblast proliferation, differentiation, and bone formation [6,7,8]. Activation of this pathway promotes the nuclear accumulation of β-Catenin, which subsequently upregulates key osteogenic genes such as Runt-related transcription factor 2 (RUNX2), thereby enhancing bone formation. Notably, the monoclonal antibody Romosozumab, which targets the Wnt signaling pathway, has been approved for the treatment of osteoporosis, further validating this pathway as a promising therapeutic target [9,10].

An increasing number of basic and clinical studies have investigated the anti-osteoporotic effects of pilose antler and its protein components. Animal studies have demonstrated that pilose antler blood preparations can significantly reverse bone loss in ovariectomized rats, increase lumbar bone mineral density (BMD), and improve trabecular microarchitecture [11]. Pilose antler peptides have been shown to upregulate osteogenic genes such as RUNX2 and Osterix (OSX) and to promote osteogenic differentiation through modulation of the Bone Morphogenetic Protein 2 and Smad signaling pathway (BMP-2/Smad) and Wnt/β-Catenin signaling pathways. In addition, aqueous extracts of pilose antler inhibit osteoclast Tartrate-resistant acid phosphatase (TRAP) activity, thereby exerting dual regulatory effects by enhancing bone formation while suppressing bone resorption [12,13]. More recently, pilose antler protein extract (PAE) has been reported to ameliorate bone loss and trabecular deterioration in ovariectomized (OVX) mouse models [14]. Furthermore, a randomized, double-blind, placebo-controlled clinical trial (*n* = 120) demonstrated that a compound preparation containing pilose antler extract and hydroxytyrosol (Ruiling capsules) significantly improved lumbar spine and femoral neck BMD, as well as bone turnover markers, in postmenopausal women after 32 weeks of treatment, without serious adverse events, indicating its potential clinical benefit in the regulation of bone metabolism [15].

Based on these findings, the present study established an ovariectomized rat model of osteoporosis and an in vitro osteoblast model to systematically evaluate the anti-osteoporotic effects of PAE. Particular emphasis was placed on elucidating whether its osteogenic activity is mediated through regulation of the Wnt/β-Catenin signaling pathway, thereby providing experimental and mechanistic evidence for the development of novel natural therapeutic candidates for osteoporosis.

## 2. Results

### 2.1. PAE Improves Femoral Bone Microarchitecture in Ovariectomized Rats

**Figure 1:**
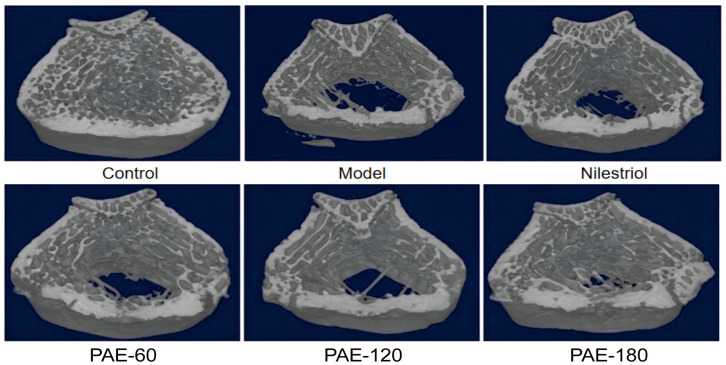
Representative three-dimensional Micro-CT reconstructions of femoral trabecular bone in ovariectomized rats: Control: sham-operated group; Model: OVX group; Nilestriol: OVX rats treated with nilestriol; PAE-60, PAE-120, and PAE-180: OVX rats treated with 60 mg/kg, 120 mg/kg and 180 mg/kg PAE, respectively.

Representative three-dimensional Micro-CT reconstructions of rat femora are shown in Figure 1. In the control group, trabecular bone exhibited a dense, continuous, and well-organized architecture, forming an intact three-dimensional network structure. In contrast, pronounced deterioration of trabecular bone was observed following ovariectomy, characterized by obvious trabecular thinning, fragmentation, and enlargement of inter-trabecular spaces, indicating severe disruption of bone microarchitecture. Administration of PAE was associated with markedly ameliorated in trabecular continuity and structural integrity. Compared with the model group, the trabecular network exhibited and better preservation in PAE-120 and PAE-180-treated rats, in which trabecular connections were partially restored. Quantitative Micro-CT analysis further supported these observations (Table 1). Ovariectomy resulted in significant reductions in BMD, Bone Volume Fraction (BV/TV), Bone Surface to Total Volume (BS/TV), Trabecular Thickness (Tb.Th), and Trabecular Number (Tb.N), accompanied by marked increases in Structure Model Index (SMI) and Trabecular Separation (Tb.Sp), indicating a more rod-like and fragile trabecular structure. PAE treatment markedly reversed these alterations. PAE-180 significantly increased BV/TV and BS/TV while reducing Tb.Sp, whereas PAE-120 significantly improved Tb.Th. Taken together, these results demonstrate that PAE treatment effectively attenuated OVX-induced deterioration of trabecular bone microarchitecture in a dose-dependent manner.

Additionally, the body weight changes throughout the experimental period were monitored and are shown in Supplementary Figure S1. The results indicate that all groups of rats exhibited weight gain as the experimental period progressed. The model group showed a faster weight increase due to ovariectomy. The Nilestriol group showed a similar weight gain trend to the control group, suggesting that the drug effectively maintained the body weight of ovariectomized rats. In the PAE groups (60 mg/kg, 120 mg/kg, 180 mg/kg), although the weight gain rate was similar to that of the model group, there were no significant differences observed between the different doses of PAE.

### 2.2. Effects of PAE on Bone Histopathological Changes

**Figure 2:**
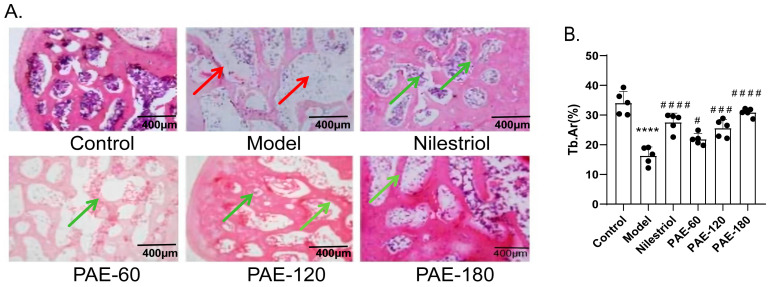
Representative hematoxylin and eosin (H&E)-stained sections and quantitative analysis of trabecular bone in the femurs of ovariectomized rats. (**A**) Control: sham-operated group; Model: ovariectomized (OVX) group; Nilestriol: OVX rats treated with nilestriol; PAE-60, PAE-120, and PAE-180: OVX rats treated with 60 mg/kg, 120 mg/kg and 180 mg/kg PAE, respectively. Red arrows indicate trabecular thinning, fragmentation, and enlarged marrow cavities in the Model group. Green arrows indicate partial restoration of trabecular structure and continuity in PAE-treated groups (PAE-60, PAE-120, PAE-180), and Nilestriol-treated group shows more significant recovery of trabecular bone structure compared to the PAE-treated groups. Scale bar = 400 μm. (**B**) Quantitative analysis of trabecular bone in the femurs of ovariectomized rats. Data are presented as mean ± SD (*n* = 5). Statistical analysis was performed using one-way ANOVA followed by Tukey’s post hoc test. **** *p* < 0.0001 vs. the control group; ^#^ *p* < 0.05, ^###^ *p* < 0.001, ^####^ *p* < 0.0001 vs. the model group. Scale bar = 200 μm.

Histopathological examination of hematoxylin and eosin (H&E)-stained femoral sections is shown in Figure 2A. In the control group, trabecular bone displayed a regular and organized architecture, with uniform thickness and clearly delineated marrow spaces. In contrast, ovariectomy induced evident structural deterioration, characterized by reduced trabecular mass, disrupted connectivity, and expanded marrow cavities, consistent with the Micro-CT findings. Treatment with PAE attenuated these histological alterations. Compared with the model group, PAE-treated rats exhibited increased trabecular preservation and improved structural continuity, accompanied by a reduction in marrow cavity enlargement. The protective effects were more pronounced in the PAE-120 and PAE-180 groups.

Quantitative analysis of trabecular area (Tb.Ar) further supported these histopathological observations. As shown in Figure 2B, the model group exhibited a significant reduction in trabecular area compared to the control group (*p* < 0.0001). Treatment with PAE significantly improved trabecular area in a dose-dependent manner. Specifically, the PAE-120 and PAE-180 groups showed a marked increase in trabecular area (*p* < 0.001), with the PAE-180 group demonstrating the most significant effect (*p* < 0.0001). Overall, these observations indicate that PAE effectively mitigated OVX-induced histopathological damage to trabecular bone.

### 2.3. Effects of PAE on Bone Metabolism and Turnover Markers

The effects of PAE on bone metabolism-related biochemical parameters and bone turnover markers are summarized in Table 2 and Table 3. As shown in Table 2, ovariectomy significantly increased serum alkaline phosphatase (ALP) activity and markly reduced bone calcium content, indicating disrupted bone metabolic homeostasis following estrogen deficiency. Similarly, bone turnover markers were significantly altered in ovariectomized rats (Table 3). Serum levels of the osteogenic markers BMP-2 and PINP were significantly decreased, whereas the bone resorption marker C-Terminal Telopeptide of Type I Collagen (CTX-I) was significantly elevated, demonstrating an imbalance between bone formation and bone resorption. PAE treatment attenuated these OVX-induced changes. PAE treatment significantly reduced serum ALP activity, suggesting partial normalization of bone metabolic activity, and significantly increased bone calcium content (Table 2). In addition, PAE administration significantly elevated serum BMP-2 and PINP levels and decreased CTX-I levels, with more pronounced effects observed in PAE-120 and PAE-180 (Table 3). Collectively, these findings suggest that PAE modulated both bone formation-related and bone resorption-related biochemical markers, contributing to the restoration of bone turnover balance in ovariectomized rats.

### 2.4. Effects of PAE on Wnt-3a and β-Catenin Expression in Femoral Bone Tissue

**Figure 3:**
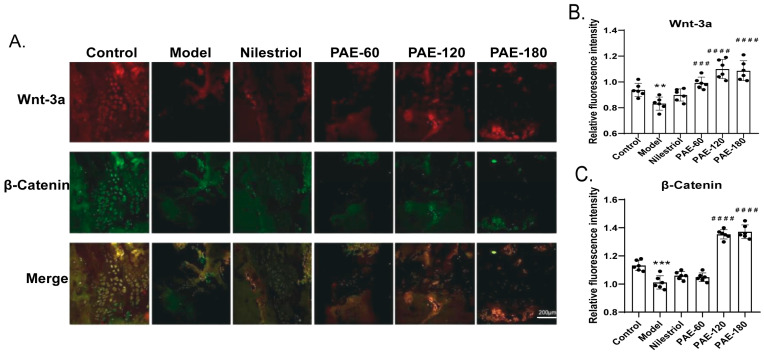
Immunofluorescence analysis of Wnt-3a and β-Catenin expression in femoral bone tissue of ovariectomized rats. (**A**) Representative immunofluorescence images showing Wnt-3a (red), β-Catenin (green), and merged signals in femoral trabecular bone from different groups. (**B**) Quantitative analysis of relative Wnt-3a fluorescence intensity. (**C**) Quantitative analysis of relative β-Catenin fluorescence intensity. Fluorescence intensity was quantified using ImageJ and analyzed with GraphPad Prism. Control: sham-operated group; Model: ovariectomized model group; Nilestriol: ovariectomized rats treated with nilestriol; PAE-60, PAE-120, and PAE-180 treated with 60 mg/kg, 120 mg/kg and 180 mg/kg PAE, respectively. Data are presented as mean ± SD (*n* = 6). Statistical analysis was performed using one-way ANOVA followed by Tukey’s post hoc test. ** *p* < 0.01, *** *p* < 0.001 vs. the control group; ^###^ *p* < 0.001, ^####^ *p* < 0.0001 vs. the model group. Scale bar = 200 μm.

The expression of Wnt-3a and β-Catenin proteins in femoral bone tissue was assessed by immunofluorescence staining and quantitative fluorescence analysis (Figure 3). Representative immunofluorescence images (Figure 3A) showed strong Wnt-3a and β-Catenin signals in the control group, whereas ovariectomy markedly reduced fluorescence intensity of in the model group. Quantitative analysis of fluorescence intensity further confirmed these findings (Figure 3B,C). Compared with the control group, Wnt-3a expression was significantly decreased in the model group (*p* < 0.01). PAE treatment significantly increased Wnt-3a expression in the PAE-60, PAE-120 and PAE-180 groups compared with the model group (*p* < 0.001). A similar trend was observed for β-Catenin expression. Ovariectomy significantly resulted β-Catenin fluorescence intensity relative to the control group. PAE-120 and PAE-180 treatment significantly increased β-Catenin expression compared with the model group (*p* < 0.001), whereas no significant difference was detected in PAE-60 group (Figure 3C). Collectively, these results indicate that PAE treatment, particularly at PAE-120 and PAE-180 doses, was associated with increased Wnt-3a and β-Catenin protein expression in femoral bone tissue.

### 2.5. Effects of PAE on Viability, Proliferation, and Osteogenic Differentiation of MC3T3-E1 Cells

**Figure 4:**
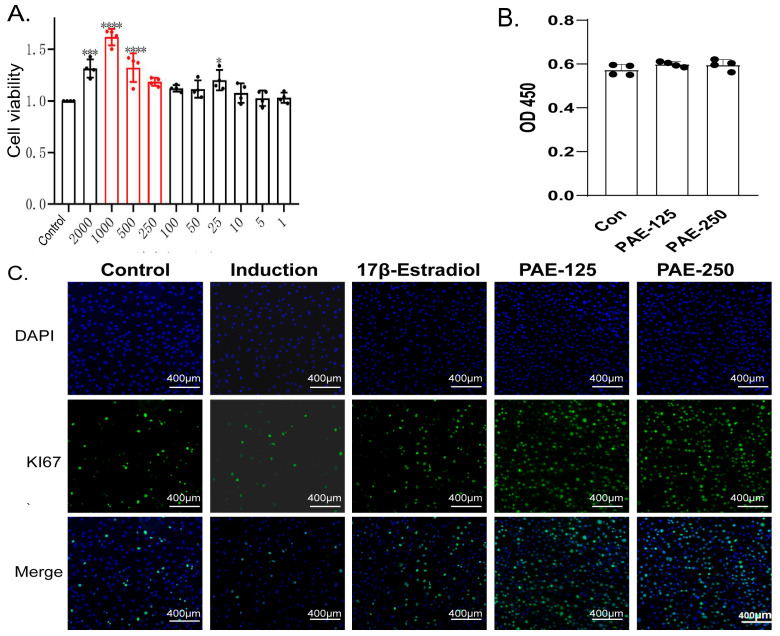

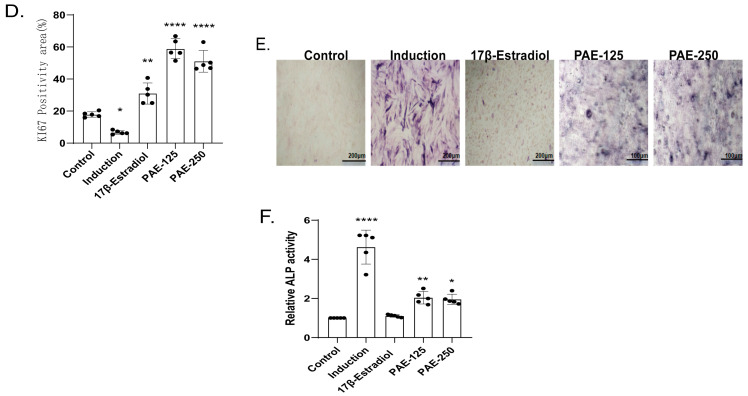
The effects of PAE on MC3T3-E1 cell viability, proliferation, and early osteogenic differentiation. (**A**) Cell viability of MC3T3-E1 cells treated with various concentrations of PAE (1–2000 μg/mL) for 24 h, as measured by the CCK-8 assay. (**B**) CCK-8 assay results for the cell-free condition. The groups included are: Control (Con) (without PAE), PAE-125 (125 μg/mL PAE), and PAE-250 (250 μg/mL PAE), *n* = 4. (**C**) Representative images of Ki67 immunofluorescence staining in MC3T3-E1 cells treated with PAE, 17β-estradiol or osteogenic induction medium. DAPI (blue) was used for nuclear staining, and Ki67-positive cells (green) indicate proliferating cells. Scale bar = 400 μm. (**D**) Quantification of Ki67-positive cells in each group. (**E**) Representative images of ALP staining in MC3T3-E1 cells treated with PAE-125, PAE-250, 17β-estradiol or osteogenic induction medium. Scale bar = 200 μm and 100 μm as indicated. (**F**) Quantitative analysis of ALP activity in MC3T3-E1 cells. Data are presented as mean ± SD (*n* = 5). Statistical analysis was performed using one-way ANOVA followed by Tukey’s post hoc test. * *p* < 0.05, ** *p* < 0.01, *** *p* < 0.001, **** *p* < 0.0001 vs. the control group.

The effects of PAE on Mouse calvarial pre-osteoblast subclone 14 (MC3T3-E1) cell viability were evaluated using the Cell Counting Kit-8 (CCK-8) assay (Figure 4A). After 24 h of treatment, PAE significantly increased cell viability within the concentration range of 100–500 μg/mL, with the most pronounced effect observed at 500 μg/mL (*p* < 0.0001), indicating a proliferative effect in this range. However, although higher concentrations (≥500 μg/mL) exhibited a strong pro-proliferative effect in the short term, such high concentrations may potentially interfere with the stability of long-term osteogenic induction, increasing the risk of false-positive differentiation responses or delayed cytotoxicity during prolonged culture. Based on these considerations, a relatively safer concentration range (125 μg/mL and 250 μg/mL) was selected for subsequent in vitro osteogenic induction experiments. To ensure no interference of PAE with the CCK-8 assay and to rule out false-positive results, testing was performed in a cell-free condition. The findings confirmed that PAE did not influence the absorbance readings of the CCK-8 reagent in the absence of cells (Figure 4B).

The effects of PAE on cell proliferation and early osteogenic differentiation are shown in (Figure 4C,D). Ki67 immunofluorescence staining demonstrated that osteogenic induction reduced the proportion of proliferating cells compared with the control group. PAE treatment enhanced this effect, which may suggest that differentiation initiation is accompanied by a decrease in proliferative capacity [16,17,18]. Specifically, the proportion of Ki67-positive nuclei was significantly higher in PAE-treated cells than in the control group and osteogenic induction medium, indicating enhanced proliferative activity during the early stage of osteogenic induction.

PAE also promoted early osteogenic differentiation. ALP staining showed the osteogenic induction of ALP activity compared with the control condition, and this effect was further enhanced by PAE treatment. Quantitative analysis confirmed that ALP activity was significantly higher in PAE-treated groups than in the control group, indicating that PAE facilitated early-stage osteogenic differentiation of MC3T3-E1 cells (Figure 4F). Collectively, these results demonstrate that PAE enhances MC3T3-E1 cell viability, proliferation, and early osteogenic differentiation.

### 2.6. Effects of PAE on Osteogenesis-Related Protein Expression and Wnt/β-Catenin Pathway Activation

**Figure 5:**
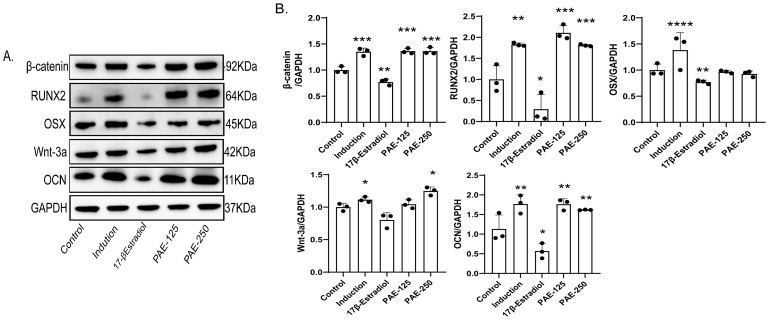

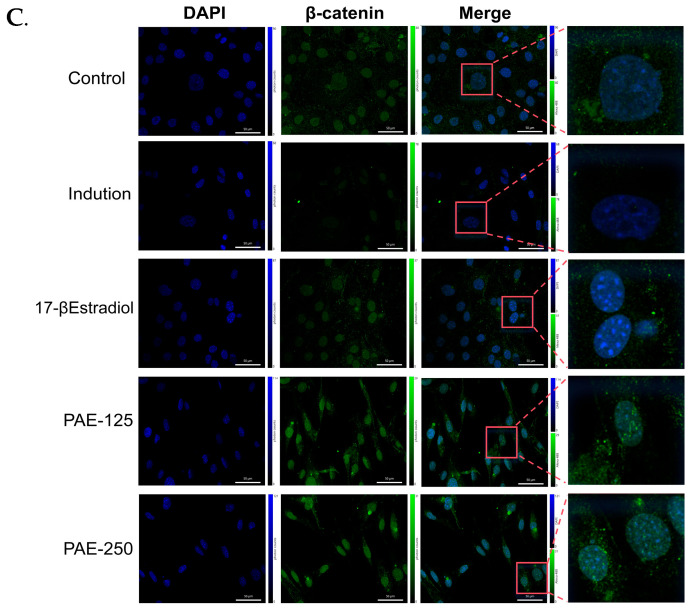
Western blot and immunofluorescence analysis of osteogenesis-related protein expression and Wnt/β-Catenin pathway activation in MC3T3-E1 cells. (**A**) Representative Western blot images showing the expression of Wnt-3a, β-Catenin, RUNX2, OSX, and OCN proteins in MC3T3-E1 cells treated with PAE, 17β-estradiol, or osteogenic induction medium. GAPDH was used as the loading control. (**B**) Quantitative analysis of relative protein expression levels. (**C**) Representative immunofluorescence images of β-Catenin staining in MC3T3-E1 cells. DAPI (blue) was used for nuclear staining, and β-Catenin (green) was visualized. Scale bar = 50 μm. Control: untreated cells; Induction: osteogenic induction medium; 17β-Estradiol: osteogenic induction with 17β-estradiol treatment; PAE-125: osteogenic induction with 125 μg/mL PAE treatment; PAE-250: osteogenic induction with 250 μg/mL PAE treatment. Data are presented as mean ± SD (*n* = 5). Statistical analysis was performed using one-way ANOVA followed by Tukey’s post hoc test. * *p* < 0.05, ** *p* < 0.01, *** *p* < 0.001, **** *p* < 0.0001 vs. the control group.

The expression of osteogenesis-related proteins and components of the Wnt/β-Catenin signaling pathway was analyzed by Western blot (Figure 5A,B). Compared with the control group, the expression of Wnt-3a was significantly increased in PAE-250 group (*p* < 0.05), while both PAE-125 and PAE-250 groups showed significantly enhanced β-Catenin expression (*p* < 0.001). These findings suggest that PAE-induced osteogenesis is accompanied by modulation of the Wnt-3a/β-Catenin pathway. In addition, the expression of key osteogenic markers, including RUNX2 and Osteocalcin (OCN), was significantly upregulated in the PAE-treated groups compared with the control group, with significant increases observed in PAE-125 and PAE-250 groups (*p* < 0.001, *p* < 0.01), reflecting the significant osteogenic effects of PAE. Notably, PAE treatment significantly upregulated the expression of the osteogenic transcription factor RUNX2 and the late-stage osteogenic marker OCN, indicating its strong osteogenic promotion effect. Although no significant changes in OSX expression were observed, it is important to note that OSX expression is phase-dependent and strongly reliant on RUNX2. Previous studies have suggested that some osteogenic pathways can bypass OSX and directly drive the expression of downstream osteogenic markers through RUNX2 [19].

Immunofluorescence staining further evaluated the activation of the Wnt/β-Catenin signaling pathway (Figure 5C). Seven days after osteogenic induction, β-Catenin expression and localization were assessed in the different groups. In the control, induction, and positive drug groups, β-Catenin signals were weak and mainly localized in the cytoplasm. In contrast, PAE-125 and PAE-250 treatment markedly enhanced β-Catenin fluorescence intensity, with increased nuclear localization. Collectively, these results demonstrate that PAE treatment enhanced osteogenesis-related protein expression and promoted activation of the Wnt/β-Catenin signaling pathway in MC3T3-E1 cells.

## 3. Discussion

Bone remodeling imbalance, characterized by uncoupled bone formation and bone resorption, represents a central pathological feature of postmenopausal osteoporosis. Estrogen deficiency accelerates osteoclastic activity while impairing osteoblastic function, ultimately leading to trabecular deterioration and bone fragility.

Restoration of this dynamic coupling is widely recognized as a key therapeutic objective in osteoporosis management [20,21,22]. In the present study, both in vivo and in vitro findings demonstrated that PAE significantly improved trabecular microarchitecture in ovariectomized rats and enhanced osteoblastic function. Mechanistically, these effects were closely associated with activation of the Wnt/β-Catenin signaling pathway, thereby providing pharmacological support for the traditional bone-strengthening properties attributed to pilose antler.

The in vivo findings revealed that PAE significantly increased BMD and BV/TV while reducing serum CTX-I levels and ALP activity in OVX rats. Estrogen replacement remains a classical intervention in ovariectomy-induced osteoporosis models due to its established anti-resorptive efficacy [23,24]. Estrogen and related analogs have been shown to regulate osteoblast and osteoclast activity, thereby contributing to the maintenance of bone mass [2]. Nilestriol, a long-acting estrogen analog, was selected as the positive control in this study because its prolonged estrogenic activity allows stable long-term administration and reduces dosing frequency in chronic OVX experiments. These changes indicate a coordinated enhancement of bone formation accompanied by suppression of excessive bone resorption. Importantly, this bidirectional modulation of bone remodeling distinguishes PAE from agents that primarily target either formation or resorption alone. Previous studies have reported that pilose antler extracts inhibit osteoclast differentiation and suppress Nuclear Factor of Activated T-cells, cytoplasmic 1 (NFATc1)-mediated transcription of osteoclast-related genes. In ANKL-induced RAW264.7 models, reductions in TRAP-positive multinucleated cells and decreased expression of Nfatc1 and Cathepsin K (Ctsk) further support an inhibitory effect on osteoclastogenesis [25]. Our findings are consistent with this regulatory profile and suggest that PAE contributes to re-establishing bone remodeling balance through coordinated control of both osteoblastic and osteoclastic activity.

Notably, despite significant increases in bone formation markers such as PINP and BMP-2, PAE did not markedly alter systemic calcium or phosphorus levels. This observation suggests that its regulatory effects may preferentially target the local bone microenvironment rather than inducing broad systemic mineral fluctuations. Such localized modulation may represent a favorable pharmacological feature, potentially reducing the risk of systemic metabolic disturbances.

At the molecular level, activation of the canonical Wnt/β-Catenin pathway emerged as a central mechanism underlying the osteogenic effects of PAE. Dose-dependent upregulation of Wnt-3a and β-Catenin expression, together with enhanced nuclear localization of β-Catenin, indicates effective pathway activation. The Wnt/β-Catenin cascade is widely recognized as a master regulator of osteoblast lineage commitment and maturation, integrating extracellular cues with transcriptional control of osteogenic genes [26,27,28]. In this study, pathway activation was accompanied by increased expression of RUNX2 and OCN, key markers of early and late osteogenic differentiation, respectively. Although OSX expression was not significantly altered, the dominant regulatory role of RUNX2 during early osteogenesis and the existence of OSX-independent differentiation routes have been previously described [16,29]. These findings suggest that PAE may preferentially amplify RUNX2-driven transcriptional programs during osteoblast differentiation.

Beyond differentiation, Wnt signaling is also implicated in the regulation of cell proliferation through modulation of cell cycle-associated genes. The increased Ki67 expression observed in PAE-treated cells is therefore consistent with enhanced proliferative activity mediated by Wnt pathway activation. This dual promotion of proliferation and differentiation is particularly relevant during early osteogenesis, when expansion of the pre-osteoblastic pool precedes matrix maturation.

The present results also resonate with current therapeutic strategies targeting Wnt signaling in osteoporosis. Romosozumab, an approved monoclonal antibody, enhances bone formation by neutralizing sclerostin, an endogenous inhibitor of Wnt signaling [30,31]. While biologic therapies have demonstrated strong anabolic efficacy, they are associated with high cost and require parenteral administration. In contrast, PAE, as a naturally derived multi-component extract, may exert modulatory effects across multiple nodes within the bone remodeling network. The capacity of PAE to simultaneously enhance osteoblast activity and restrain bone resorption suggests a broader regulatory spectrum compared with single-target agents. Furthermore, clinical evidence indicating improved BMD and bone turnover markers in postmenopausal women receiving pilose antler protein extract-based formulations [15] supports the translational potential of this approach.

Nevertheless, several limitations should be considered. Although Wnt pathway modulation was observed in the present study, a direct causal relationship cannot be definitively established due to the lack of pathway-specific inhibition or genetic interference experiments. Additionally, mineralization assays (e.g., Alizarin Red or Von Kossa staining) were not performed, which may limit the evaluation of late-stage osteogenic maturation. Future studies integrating transcriptomic or proteomic profiling, along with comprehensive mineralization assessments, could provide a deeper understanding of PAE-mediated signaling networks. Furthermore, the impact of PAE on mitochondrial activity was not directly assessed in this study, and future research should explore whether PAE influences mitochondrial function, particularly in relation to osteogenesis. Finally, long-term efficacy and safety assessments in extended animal models are needed before progressing toward clinical application. Additionally, considering the potential clinical application of PAE-based therapies for osteoporosis in humans would be a valuable direction for future research, with an emphasis on identifying the feasibility and therapeutic benefits of such treatments in clinical practice.

## 4. Materials and Methods

### 4.1. Animals and Cells Female

Female Wistar rats (8–10 weeks old, 200–220 g) were purchased from Beijing Vital River Laboratory Animal Technology Co., Ltd. (License No. SCXK (Beijing, China) 2016-0011). Animals were housed under controlled conditions with free access to food and water. All experimental procedures were approved by the Ethics Committee of the Chinese Academy of Medical Sciences (Ethical Approval No. ERCCACMS21-2506-01).

MC3T3-E1 Subclone 14 pre-osteoblastic cells were obtained from Wuhan Procell Life Science & Technology Co., Ltd., Wuhan, China (Catalog No. CL-0378).

### 4.2. Extraction and Characterization of Pilose Antler Protein

Fresh pilose antler (Yunlu Group, Changchun, China) was mechanically crushed and homogenized into a paste, The material was extracted overnight at 4 °C with distilled water containing glacial acetic acid. The mixture was centrifuged (5000 rpm, 10 min, 4 °C) to collect the supernatant, which was freeze-dried. The yield was calculated, and the powder was stored at 4 °C.

To ensure the quality and consistency of PAE, we conducted a detailed proteomic analysis in our previous study [32]. This study demonstrated that the major protein molecular weight range of PAE is 35–140 kDa, the highest intensity was detected near 140 kDa. LC-MS further confirmed the proteomic characteristics of PAE and revealed its batch-to-batch consistency. The mass spectrometry data of PAE have been submitted to ProteomeXchange (data accession number: PXD037328). The Liquid Chromatography-Mass Spectrometry (LC-MS) data and Sodium Dodecyl Sulfate Polyacrylamide Gel Electrophoresis (SDS-PAGE) images have been uploaded as Supplementary Materials Table S1 and Figure S2 to support the quality control and standardization of PAE.

### 4.3. Reagents and Antibodies

All reagents, unless otherwise specified, were purchased from Sigma-Aldrich (St. Louis, MO, USA). All primary and secondary antibodies used in this study were obtained from Abcam (Cambridge, UK). The primary antibodies included anti-Wnt-3a (Cat. No. ab219412), anti-β-Catenin (Cat. No. ab32572), anti-RUNX2 (Cat. No. ab192256), anti-OCN (Cat. No. ab309521), anti-OSX (Cat. No. ab209484), anti-Ki67 (Cat. No. ab16667), and anti-GAPDH (Cat. No. ab181602). For Western blot analysis, Goat anti-rabbit IgG H&L (HRP) (Cat. No. ab6721) was used. For immunofluorescence staining, Alexa Fluor-conjugated goat anti-rabbit IgG secondary antibodies (Alexa Fluor^®^ 488, Cat. No. ab150077; Alexa Fluor^®^ 594, Cat. No. ab150080, Thermo Fisher Scientific, Waltham, MA, USA) were applied.

### 4.4. Animal Grouping and Treatment

Animals were randomly assigned using a computer-generated randomization list. A total of 36 female Wistar rats were randomly assigned to six groups (*n* = 6 per group): control, model (OVX), positive drug, and PAE-60, PAE-120, and PAE-180 groups. Osteoporosis was induced by bilateral OVX in all groups except the control group, which underwent sham surgery. Treatments were administered by oral gavage once daily for 13 weeks. The control and model groups received saline (10 mL/kg). The positive drug group received Nilestriol (3.6 mg/kg) (Shanghai Xinhualian Pharmaceutical Co., Ltd., No. H31021647, Shanghai, China). This dosage has already passed safety evaluation in our preliminary experiments. The body weight of the rats was recorded weekly throughout the 13-week experimental period. The PAE-60, PAE-120, and PAE-180 groups received pilose antler protein extract at doses of 60, 120, and 180 mg/kg. The selected doses (60–180 mg/kg) were established based on preliminary dose-finding experiments evaluating safety and efficacy trends in OVX rats. These doses were well tolerated and showed preliminary indications of osteoprotective activity, and were therefore chosen for formal pharmacological evaluation. Investigators performing Micro-CT analysis, histological evaluation, and biochemical measurements were blinded to group allocation.

### 4.5. Cell Culture and Treatment

MC3T3-E1 Subclone 14 cells were seeded in 96-well plates and treated with deer m antler protein extract at concentrations of 2000, 1000, 500, 250, 100, 50, 25, 10, 5, 2.5, and 1 μg/mL for 24 h. Cell viability was assessed using the CCK-8 assay, and absorbance was measured at 450 nm. For osteogenic differentiation, cells were cultured in osteogenic induction medium supplemented with either 17β-estradiol (100 nM) (Selleck Chemicals LLC, catalog no. S1709, Houston, TX, USA), or PAE (125 μg/mL and 250 μg/mL). The medium was refreshed every two days.

### 4.6. MicroCT Measurement of Femoral Bone Density in Rats

After 13 weeks, femora were harvested, fixed in 4% paraformaldehyde and scanned using a MicroCT system (SkyScan 1276, Bruker, Kontich, Belgium). Parameters including bone mineral density (BMD), bone volume fraction (BV/TV), trabecular thickness (Tb.Th), and other parameters were analyzed.

### 4.7. ELISA Measurement of BMP-2, PINP, and CTX I Levels in Serum

Serum levels of BMP-2, PINP, and CTX-I were measured using commercial ELISA kits according to the manufacturers’ instructions (BMP-2: Catalog No. USEA013Ra; PINP: Catalog No. UCEA957Ra; CTX-I: Catalog No. UCEA665Ra; Cloud-Clone Technology Co., Ltd., Wuhan, China). Serum samples were centrifuged at 3000 rpm for 10 min and stored at −80 °C until analysis. For the PINP measurement, serum samples from the PAE-120 and PAE-180 groups were subjected to a 2-fold dilution prior to analysis. All experiments were performed in duplicate to ensure the reliability and reproducibility of the results.

### 4.8. Chemical Assay for Calcium, Phosphate Content, and Alkaline Phosphatase Activity

Serum calcium levels were determined using a colorimetric calcium assay kit (Catalog No. MAK022, Sigma-Aldrich, St. Louis, MO, USA), based on the o-cresolphthalein complexone method. Serum phosphorus concentrations were measured using a colorimetric phosphate assay kit (Catalog No. MAK030, Sigma-Aldrich, St. Louis, MO, USA), based on the phosphomolybdate colorimetric method. Absorbance was measured at the recommended wavelengths using a microplate reader, and concentrations were calculated from standard calibration curves. Serum alkaline phosphatase (ALP) activity was determined using an ALP activity assay kit (Catalog No. 21101ES60, Yeason Biotechnology Shanghai Co., Ltd., Shanghai, China), based on the colorimetric detection of p-nitrophenol generated from the hydrolysis of p-nitrophenyl phosphate (pNPP).

For the rat femur acid digestion, the femur was separated and cleaned of all soft tissues and bone marrow, rinsed with PBS, and dried in an oven at 80 °C for 72 h. The dry femur weight was measured, and 0.5 mL of 6 mol/L HCl was added for acid digestion, which was incubated at 95 °C for 24 h. After centrifuging at 12,000 rpm for 10 min, the supernatant was collected and diluted at a ratio of 1:500 before analysis. Serum calcium samples were diluted 3-fold, and phosphate samples were diluted 50-fold. ALP samples were used without dilution.

### 4.9. HE Staining for Histopathological Examination of Bone Tissue

Femora were decalcified, dehydrated, embedded in paraffin, and sectioned into 5 μm thick slices, stained with hematoxylin and eosin (HE), and analyzed using the RVL2-K2 Inverted and Upright Microscope Imaging System (ECHO, Lake Zurich, IL, USA).

### 4.10. Immunofluorescence Analysis of Wnt and β-Catenin Expression in Tissue

Paraffin sections were deparaffinized and incubated overnight at 4 °C with primary antibodies against Wnt-3a (1:100 dilution) and β-Catenin (1:200 dilution). After primary antibody incubation, the sections were incubated with the appropriate fluorescence-conjugated secondary antibodies: goat anti-rabbit IgG Alexa Fluor^®^ 594 for Wnt-3a (red fluorescence) and goat anti-rabbit IgG Alexa Fluor^®^ 488 for β-Catenin (green fluorescence). Images were captured under a fluorescence microscope (200× magnification). Integrated optical density (IOD) was analyzed with ImageJ-Fiji 1.54f software.

### 4.11. Immunofluorescence Detection of Ki67 and β-Catenin Expression in Cells

MC3T3-E1 cells were incubated with primary antibodies against Ki67 (1:200 dilution) and β-Catenin (1:200 dilution) overnight at 4 °C, followed by fluorescence-conjugated secondary antibodies (1:500 dilution) and DAPI nuclear staining. Fluorescence intensity and ki67-positive cells were quantified using ImageJ software.

### 4.12. BCIP/NBT Alkaline Phosphatase (ALP) Staining

Cells were fixed and incubated with BCIP/NBT staining solution (Beyotime Biotechnology Co., Ltd., Catalog No. C3206, Shanghai, China) for 1 h. ALP activity was observed under a light microscope and analyzed using ImageJ software.

### 4.13. Western Blot Analysis of Wnt-3a, β-Catenin, RUNX2, OCN, and OSX Protein Expression

Total protein was extracted and quantified using the BCA assay. Proteins were separated by SDS-PAGE and transferred to PVDF (Catalog No. IPVH00010, Millipore, Darmstadt, Germany) membranes. Membranes were incubated overnight at 4 °C with primary antibodies, i.e., Wnt-3a (1:1000 dilution), β-Catenin (1:5000 dilution), RUNX2 (1:1000 dilution), OCN (1:1000 dilution), OSX (1:1000 dilution) and GAPDH (1:1000 dilution), followed by HRP-conjugated secondary antibodies (1:10,000 dilution). Protein bands were visualized using a chemiluminescent imaging system (JY04S-3C, Beijing Junyi Dongfang Electrophoresis Equipment Co., Ltd., Beijing, China) and quantified using ImageJ software.

### 4.14. Statistical Analysis

Date are presented as mean ± SD. Statistical comparison were performed using the one-way analysis of variance (ANOVA) followed by Tukey’s post hoc text. Statistical analyses were conducted using GraphPad Prism version 8.0 software. A *p*-value of <0.05 was considered statistically significant.

## 5. Conclusions

In summary, PAE promotes osteoblast proliferation and differentiation, improves trabecular microarchitecture, and suppresses bone resorption in ovariectomized rats. These effects are mediated, at least in part, through activation of the Wnt/β-Catenin signaling pathway. By restoring the balance between bone formation and resorption, PAE demonstrates multi-target and dual-regulatory properties that support its development as a natural therapeutic candidate for osteoporosis. Future studies focusing on active component isolation, precise molecular target identification, and comprehensive preclinical evaluation will further facilitate its translational advancement.

## Figures and Tables

**Table 1:** Quantitative Micro-CT analysis of femoral trabecular bone microarchitecture in ovariectomized rats.

Group	BMD, g/cm^3^	BV/TV, %	BS/TV, 1/μm	SMI	Tb.Th, μm	Tb.N, 1/μm	Tb.Sp, μm
Control	0.0104 ± 0.002	39.94 ± 3.12	0.0112 ± 0.0020	0.484 ± 0.112	108.7 ± 3.19	0.0037 ± 0.0004	237.2 ± 78.2
Model	0.0041 ± 0.001 ***	10.83 ± 2.92 **	0.0041 ± 0.0010 **	2.340 ± 0.256 **	93.29 ± 4.16 **	0.0011 ± 0.0003 **	941.9 ± 296.6 **
Nilestriol	0.0045 ± 0.001	12.17 ± 4.19	0.0045 ± 0.0014	1.725 ± 0.357 ^#^	95.67 ± 6.6	0.0013 ± 0.0005	854.5 ± 106.8
PAE-60	0.0056 ± 0.002	14.71 ± 2.45	0.0056 ± 0.0015	1.820 ± 0.402	100.4 ± 4.16	0.0014 ± 0.0003	454.7 ± 262.5 ^#^
PAE-120	0.0056 ± 0.002	13.2 ± 2.42	0.0055 ± 0.0025	1.898 ± 0.280	106.3 ± 7.42 ^#^	0.0012 ± 0.0002	498.9 ± 220.13 ^#^
PAE-180	0.0064 ± 0.002	15.93 ± 1.78 ^#^	0.0064 ± 0.0017 ^#^	1.994 ± 0.324	97.92 ± 5.3	0.0016 ± 0.0003	338.7 ± 248.7 ^##^

Data are presented as mean ± SD (*n* = 6). BMD, bone mineral density; BV/TV, bone volume fraction; BS/TV, bone mineral density; Tb.Th, trabecular thickness; Tb.N, trabecular number; Tb.Sp, trabecular separation; SMI, structure model index. ** *p* < 0.01, *** *p* < 0.001 vs. the control group; ^#^ *p* < 0.05, ^##^ *p* < 0.01 vs. the model group.

**Table 2:** Effects of PAE on serum biochemical parameters and bone calcium content.

Group	Serum Ca (nmol/μL)	Serum P (nmol/μL)	ALP (U/L)	Bone Ca (mg/g)
Control	2.111 ± 0.203	1.037 ± 0.173	65.98 ± 10.01	247.08 ± 36.29
Model	1.741 ± 0.275 **	0.945 ± 0.187	107.74 ± 20.66 ****	184.22 ± 21.54 ****
Nilestriol	1.768 ± 0.337	0.926 ± 0.136	76.67 ± 17.90 ^###^	219.38 ± 30.58 ^#^
PAE-60	1.907 ± 0.327	0.966 ± 0.178	80.99 ± 15.82 ^##^	221.21 ± 26.39 ^#^
PAE-120	2.083 ± 0.324 ^#^	1.135 ± 0.199	81.74 ± 17.20 ^##^	217.06 ± 30.32 ^#^
PAE-180	2.080 ± 0.163 ^#^	1.125 ± 0.220	81.00 ± 18.78 ^##^	216.38 ± 29.17 ^#^

Data are presented as mean ± SD (*n* = 6). Ca, calcium; P, phosphorus; ALP, alkaline phosphatase. Statistical analysis was performed using one-way ANOVA followed by Dunnett’s multiple comparisons test. ** *p* < 0.01, **** *p* < 0.001 vs. the control group; ^#^ *p* < 0.05, ^##^ *p* < 0.01, ^###^ *p* < 0.001, vs. the model group.

**Table 3:** Effects of PAE on serum bone turnover markers in ovariectomized rats.

Group	BMP-2 (pg/mL)	PINP (ng/mL)	CTX I (pg/mL)
Control	257.48 ± 20.87	15.84 ± 2.23	78.89 ± 32.23
Model	157.79 ± 18.05 ****	11.20 ± 2.26 *	597.33 ± 190.15 ****
Nilestriol	211.18 ± 12.61 ^####^	13.91 ± 2.86	415.73 ± 168.35 ^#^
PAE-60	185.44 ± 14.60 ^##^	16.04 ± 2.09 ^##^	580.88 ± 164.62
PAE-120	204.46 ± 17.77 ^####^	24.29 ± 5.67 ^####^	343.12 ± 104.21 ^###^
PAE-180	211.46 ± 20.30 ^####^	25.11 ± 5.68 ^####^	301.20 ± 137.36 ^####^

Data are presented as mean ± SD (*n* = 6). BMP-2, bone morphogenetic protein 2; PINP, procollagen type I N-terminal propeptide; CTX-I, C-terminal telopeptide of type I collagen. Statistical analysis was performed using one-way ANOVA followed by Dunnett’s multiple comparisons test. * *p* < 0.05, **** *p* < 0.0001 vs. the control group; ^#^ *p* < 0.05, ^##^ *p* < 0.01, ^###^ *p* < 0.001, ^####^ *p* < 0.0001 vs. the model group.

## Data Availability

The original contributions presented in this study are included in the article/Supplementary Material. Further inquiries can be directed to the corresponding author.

## Supplementary Materials

The following supporting information can be downloaded at: https://www.mdpi.com/article/10.3390/ph19050665/s1. Figure S1: Body weight changes of experimental animals throughout the experimental period. Body weights were measured weekly for 13 weeks. All groups showed steady weight gain during the experimental period, and the model group had the fastest weight growth. Figure S2: SDS-PAGE gel electrophoresis of total proteins from PAE. Total proteins were extracted from six independent batches of PAE(PAE1-PAE6), and total protein detection was performed under unified experimental conditions. The highest expression of PAE proteins is observed at approximately 140 kDa. Table S1: LC-MS analysis of PAE. The table displays all high-confidence master proteins identified from PAE via LC-MS/MS detection, including protein accession numbers, functional annotation information, characteristic peptide sequences, and scaled relative abundance data of each protein.

## References

[1] Kanis J.A., Cooper C., Rizzoli R., Reginster J.Y. (2018). European Guidance for the Diagnosis and Management of Osteoporosis in Postmenopausal Women. Osteoporos Int..

[2] Compston J., McClung M.R., Leslie W.D. (2019). Osteoporosis. Lancet.

[3] Li C. (2023). Deer antler renewal gives insights into mammalian epimorphic regeneration. Cell Regen..

[4] Wang G., Meng Y., Ouyang W., Zhao C., Zhao W. (2023). Effect of pilose antler polypeptide on the mechanism of bone homeostasis in osteoporosis. Front. Med..

[5] Sun H., Xiao D., Liu W., Li X., Lin Z., Li Y., Ding Y. (2024). Well-known polypeptides of deer antler velvet with key actives: Modern pharmacological advances. Naunyn Schmiedebergs Arch. Pharmacol..

[6] Wang X.H., Qu Z.C., Zhao S.C., Luo L., Yan L. (2024). Wnt/β-Catenin Signaling Pathway: Proteins’ Roles in Osteoporosis and Cancer Diseases and the Regulatory Effects of Natural Compounds on Osteoporosis. Mol. Med..

[7] Hu L., Chen W., Qian A., Li Y.-P. (2024). Wnt/β-Catenin Signaling Components and Mechanisms in Bone Formation, Homeostasis, and Disease. Bone Res..

[8] Rivadeneira F., Mäkitie O. (2021). Osteoporosis and Bone Mass Disorders: From Gene Pathways to Treatments. Nat. Rev. Endocrinol..

[9] McClung M.R., Betah D., Leder B.Z., Kendler D.L., Oates M., Timoshanko J., Wang Z. (2025). Romosozumab Improves Microarchitecture as Assessed by Tissue Thickness-Adjusted Trabecular Bone Score in Postmenopausal Women with Osteoporosis. J. Bone Miner. Res..

[10] Wu D., Li L., Wen Z., Wang G. (2023). Romosozumab in Osteoporosis: Yesterday, Today, and Tomorrow. J. Transl. Med..

[11] Wang T., Luo E., Zhou Z., Yang J., Wang J., Zhong J., Zhang J., Yao B., Li X., Dong H. (2023). Lyophilized powder of velvet antler blood improves osteoporosis in OVX-induced mouse model and regulates proliferation and differentiation of primary osteoblasts via Wnt/β-Catenin pathway. J. Funct. Foods.

[12] Ho T.J., Tsai W.T., Wu J.R., Chen H.P. (2024). Biological activities of deer antler-derived peptides on human chondrocyte and bone metabolism. Pharmaceuticals.

[13] Ren C., Gong W., Li F., Xie M. (2019). Pilose antler aqueous extract promotes the proliferation and osteogenic differentiation of bone marrow mesenchymal stem cells by stimulating the BMP-2/Smad1,5/Runx2 signaling pathway. Chin. J. Nat. Med..

[14] Pan W., Du J., An L., Xu G., Yuan G., Sheng Y., Sun J., Wang M., Zhao N., Guo X. (2023). Sika deer velvet antler protein extract modulates bone metabolism and the structure of gut microbiota in ovariectomized mice. Food Sci. Nutr..

[15] Ma X., Ma Y., Ma X., Zhang Z., Li Y. (2025). Combination of pilose antler extract and hydroxytyrosol enhances bone mineral density in both animals and postmenopausal women. Food Sci. Nutr..

[16] Ponzetti M., Rucci N. (2021). Osteoblast differentiation and signaling: Established concepts and emerging topics. Int. J. Mol. Sci..

[17] Xu W., Li Y., Feng R., He P., Zhang Y. (2022). γ-Tocotrienol induced the proliferation and differentiation of MC3T3-E1 cells through stimulation of the Wnt/β-Catenin signaling pathway. Food Funct..

[18] Jiang Q., Nagano K., Moriishi T., Komori H., Sakane C., Matsuo Y., Zhang Z., Nishimura R., Ito K., Qin X. (2024). Roles of Sp7 in osteoblasts for the proliferation, differentiation, and osteocyte process formation. J. Orthop. Transl..

[19] Zhu S., Chen W., Masson A., Li Y.-P. (2024). Cell Signaling and Transcriptional Regulation of Osteoblast Lineage Commitment, Differentiation, Bone Formation, and Homeostasis. Cell Discov..

[20] Bolamperti S., Villa I., Rubinacci A. (2022). Bone remodeling: An operational process ensuring survival and bone mechanical competence. Bone Res..

[21] Durdan M.M., Azaria R.D., Weivoda M.M. (2022). Novel insights into the coupling of osteoclasts and resorption to bone formation. Semin. Cell Dev. Biol..

[22] McNamara L.M. (2021). Osteocytes and estrogen deficiency. Curr. Osteoporos. Rep..

[23] Yousefzadeh N., Kashfi K., Jeddi S., Ghasemi A. (2020). Ovariectomized rat model of osteoporosis: A practical guide. EXCLI J..

[24] Riggs B.L., Khosla S., Melton L.J. (2002). Sex steroids and the construction and conservation of the adult skeleton. Endocr. Rev..

[25] Choi Y.Y., Jin S.C., Song M., Yi S., Park J., Baek H.K., Park S.H., Yang H.J., Lee J.Y., Yang W.M. (2026). Cervus elaphus sibiricus (deer antler) extract alleviates osteoporosis via dual modulation of osteoblast and osteoclast activity in ovariectomy-induced mice based on network pharmacology. J. Ethnopharmacol..

[26] Vlashi R., Zhang X., Wu M., Chen G. (2023). Wnt signaling: Essential roles in osteoblast differentiation, bone metabolism and therapeutic implications for bone and skeletal disorders. Genes Dis..

[27] Wróbel E., Wojdasiewicz P., Mikulska A., Szukiewicz D. (2025). β-Catenin: A key molecule in osteoblast differentiation. Biomolecules.

[28] Kobayashi Y., Iwamoto R., He Z., Udagawa N. (2025). Wnt family members regulating osteogenesis and their origins. J. Bone Miner. Metab..

[29] Komori T. (2024). Regulation of skeletal development and maintenance by Runx2 and Sp7. Int. J. Mol. Sci..

[30] Mäkinen V.N., Sølling A.S., McClung M., Langdahl B.L. (2025). Romosozumab for the treatment of osteoporosis—A systematic review. J. Endocrinol. Investig..

[31] Marini F., Giusti F., Palmini G., Brandi M.L. (2023). Role of Wnt signaling and sclerostin in bone and as therapeutic targets in skeletal disorders. Osteoporos. Int..

[32] Li L., Wang Y., Ma Y., Gong J., Ren X., Sun Y. (2025). Pilose Antler Extract Promotes Angiogenesis and Vascular Maturation to Accelerate Wound Healing. Sci. Rep..

