# Supplementary figures and images for: Pilose Antler Protein Extract Alleviates Osteoporosis and Is Associated with Activation of the Wnt/β-Catenin Signaling Pathway
Source: Pharmaceuticals (Basel). 2026 Apr 24;19(5):665. doi: 10.3390/ph19050665 (PMC13210146; doi:10.3390/ph19050665)

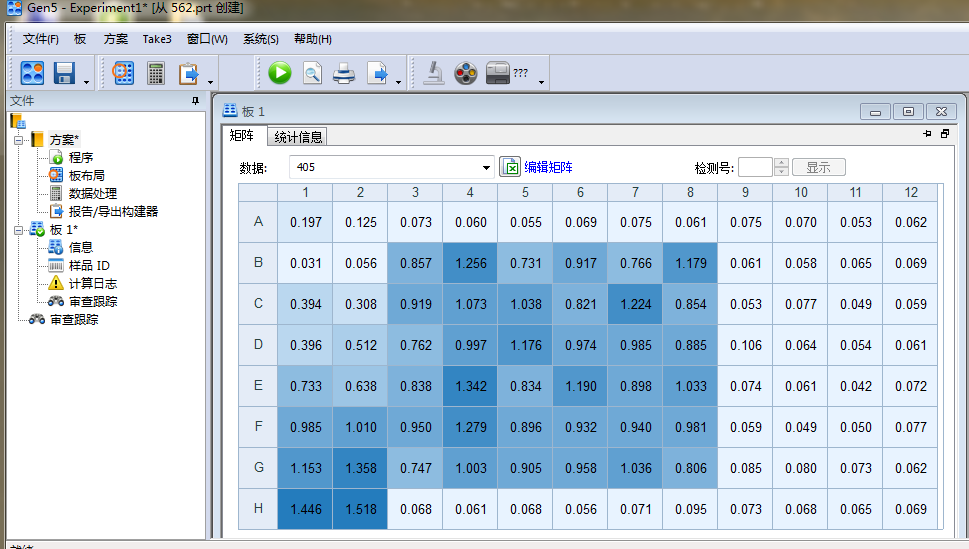

Supplement: Supplementary file 1 [file pharmaceuticals-19-00665-s001.zip › Data--Please check if this file does not need to be published/raw data/ALP/ALP-1.PNG]

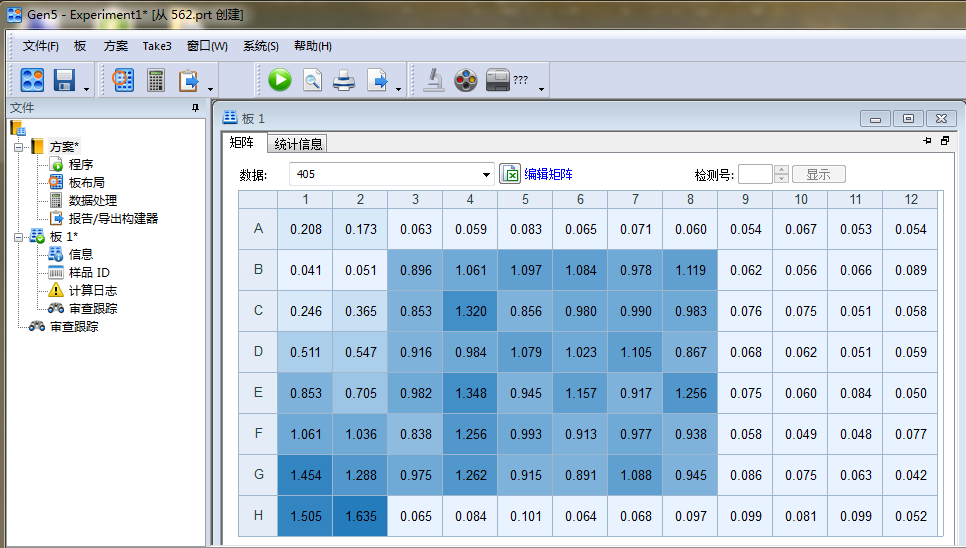

Supplement: Supplementary file 1 [file pharmaceuticals-19-00665-s001.zip › Data--Please check if this file does not need to be published/raw data/ALP/alp-2.PNG]

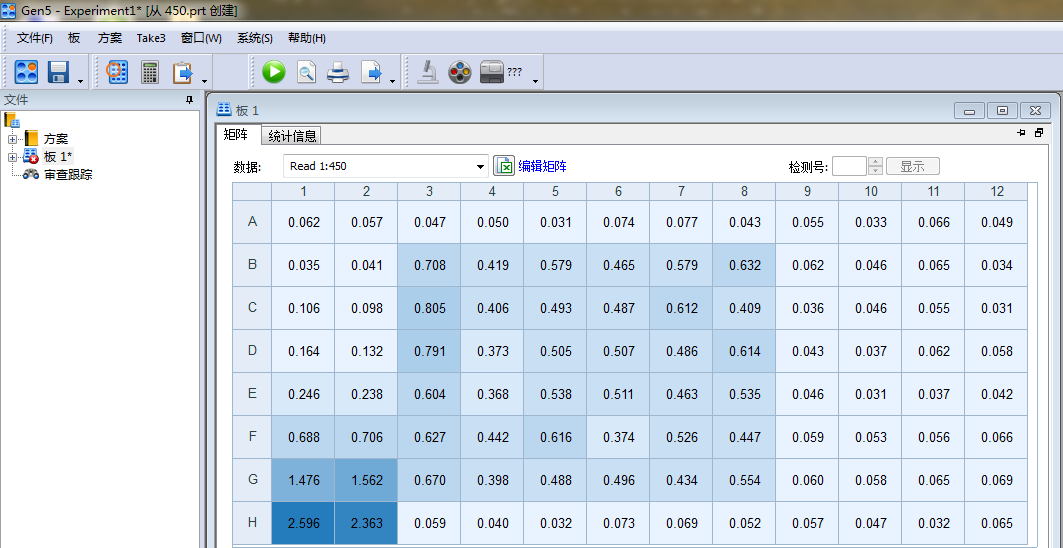

Supplement: Supplementary file 1 [file pharmaceuticals-19-00665-s001.zip › Data--Please check if this file does not need to be published/raw data/bmp2/BMP-2-1.PNG]

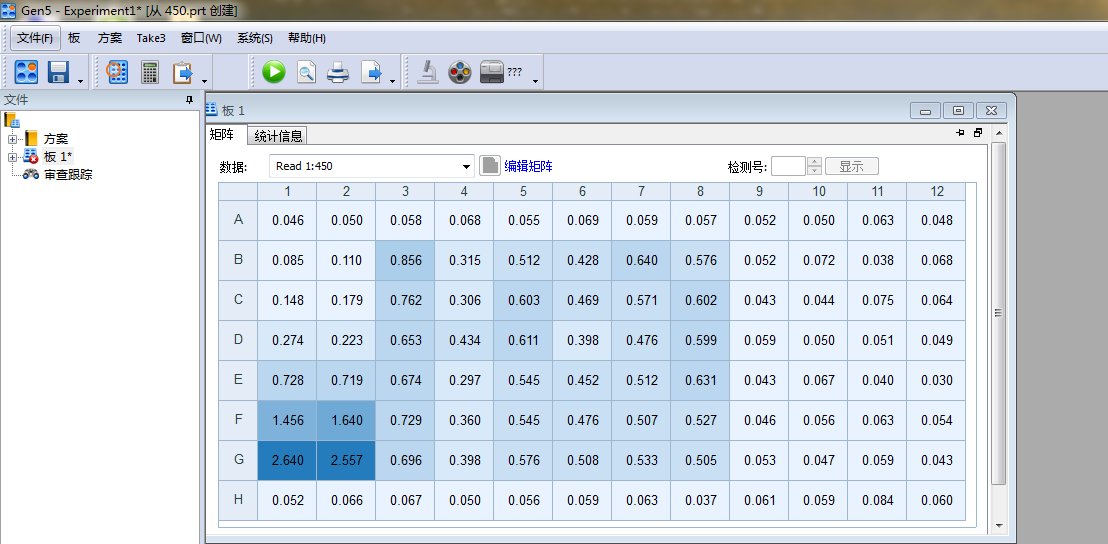

Supplement: Supplementary file 1 [file pharmaceuticals-19-00665-s001.zip › Data--Please check if this file does not need to be published/raw data/bmp2/BMP-2-2.PNG]

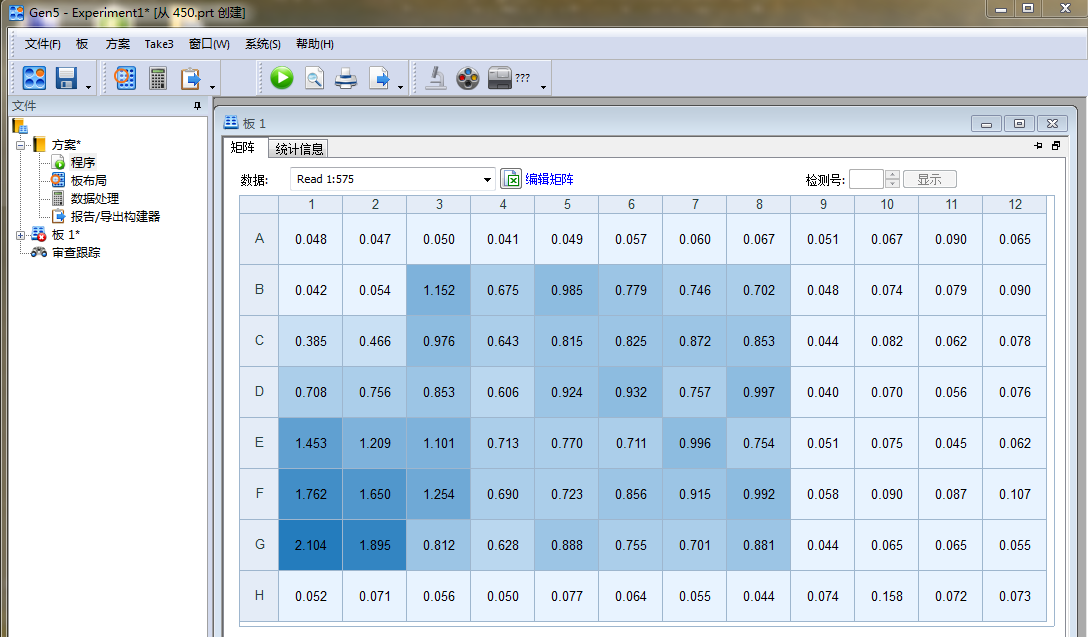

Supplement: Supplementary file 1 [file pharmaceuticals-19-00665-s001.zip › Data--Please check if this file does not need to be published/raw data/bone-Ca/bone-Ca-1.PNG]

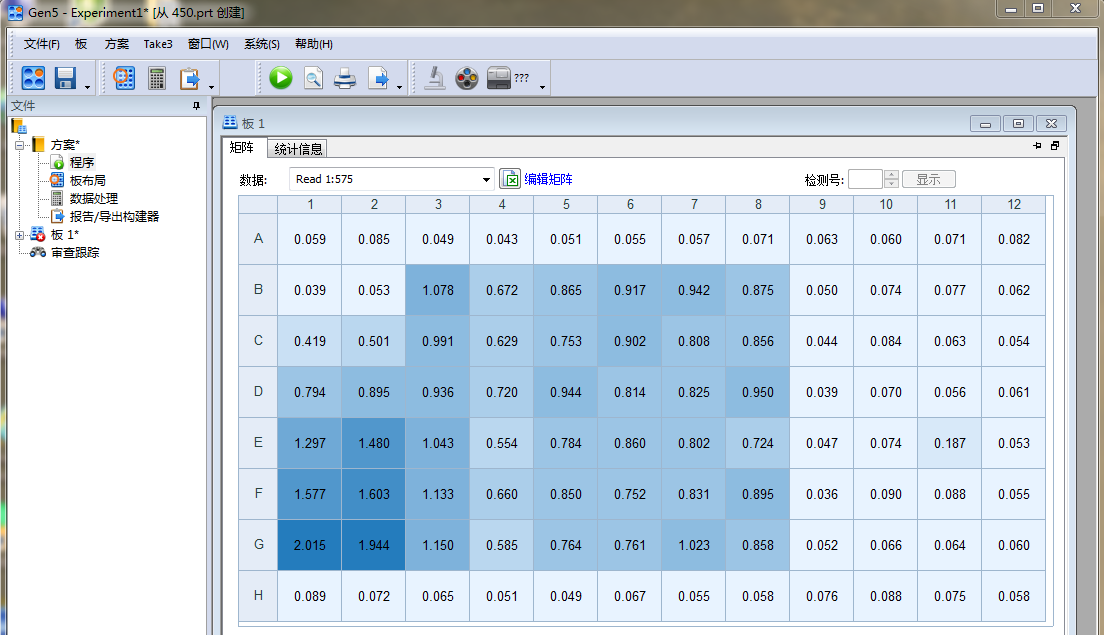

Supplement: Supplementary file 1 [file pharmaceuticals-19-00665-s001.zip › Data--Please check if this file does not need to be published/raw data/bone-Ca/bone-Ca-2.PNG]

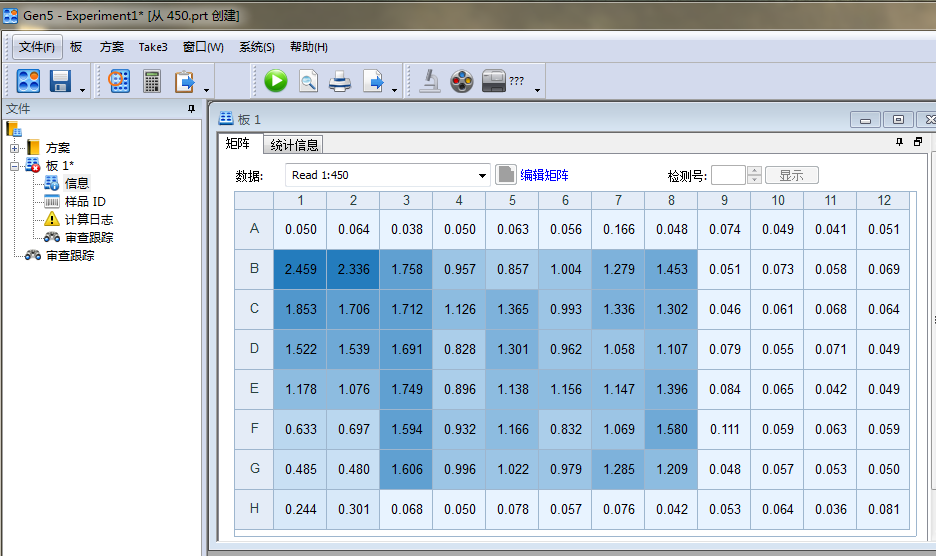

Supplement: Supplementary file 1 [file pharmaceuticals-19-00665-s001.zip › Data--Please check if this file does not need to be published/raw data/ctx-1/ctx-1-1.PNG]

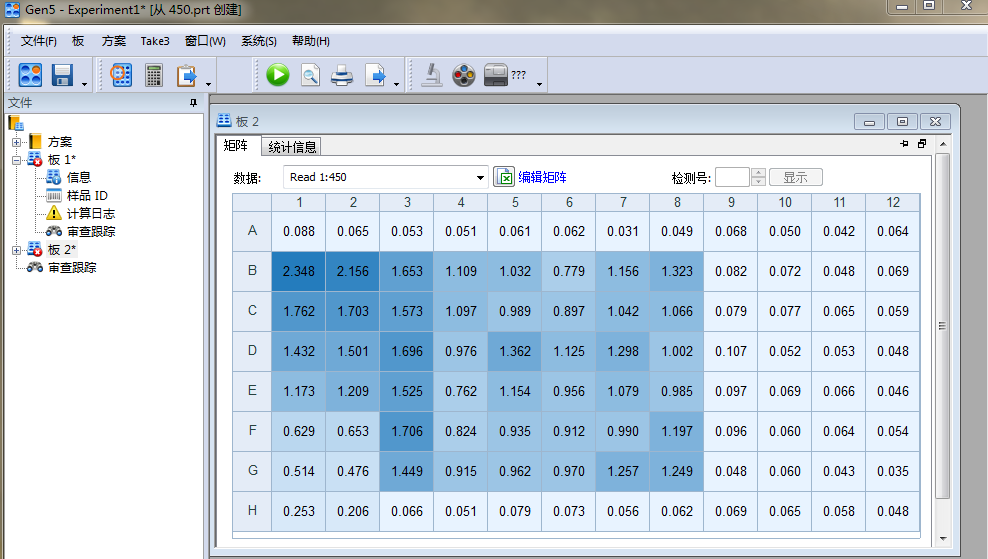

Supplement: Supplementary file 1 [file pharmaceuticals-19-00665-s001.zip › Data--Please check if this file does not need to be published/raw data/ctx-1/ctx-1-2.PNG]

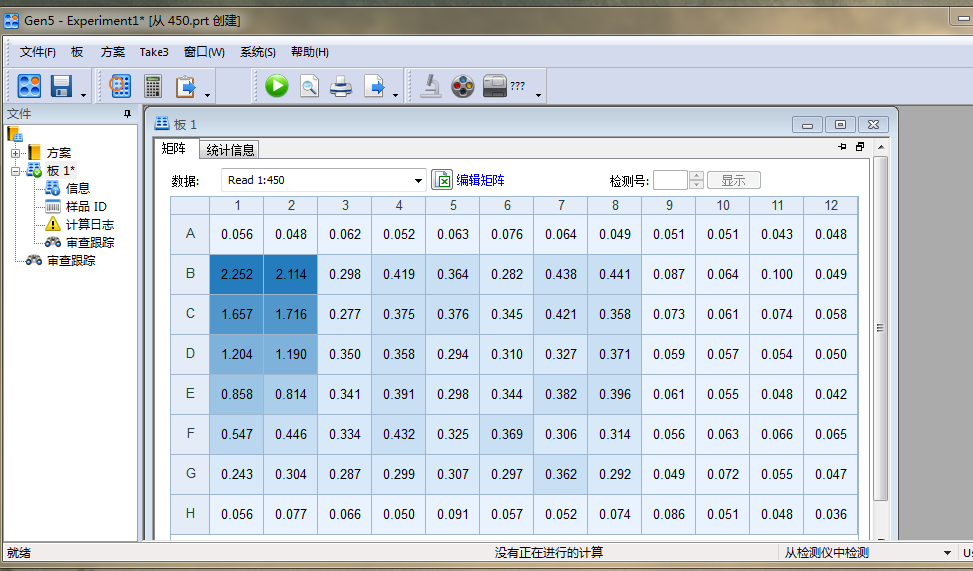

Supplement: Supplementary file 1 [file pharmaceuticals-19-00665-s001.zip › Data--Please check if this file does not need to be published/raw data/pinp/pinp-1-1.PNG]

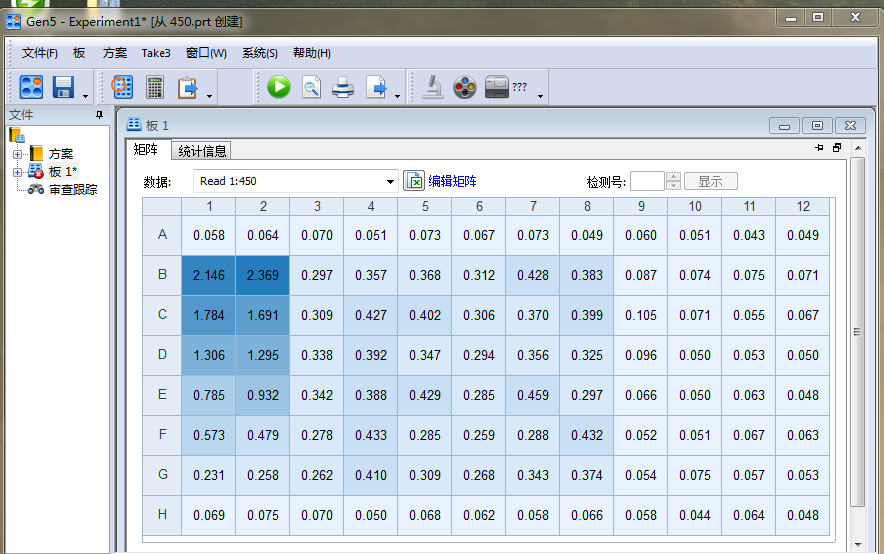

Supplement: Supplementary file 1 [file pharmaceuticals-19-00665-s001.zip › Data--Please check if this file does not need to be published/raw data/pinp/pinp-2.PNG]

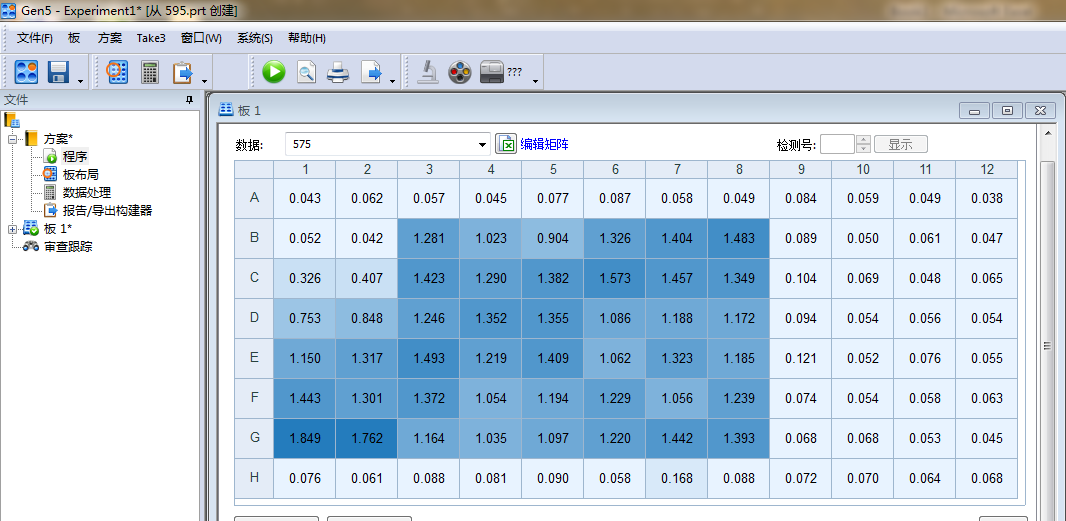

Supplement: Supplementary file 1 [file pharmaceuticals-19-00665-s001.zip › Data--Please check if this file does not need to be published/raw data/serum-ca/serum-Ca-1.PNG]

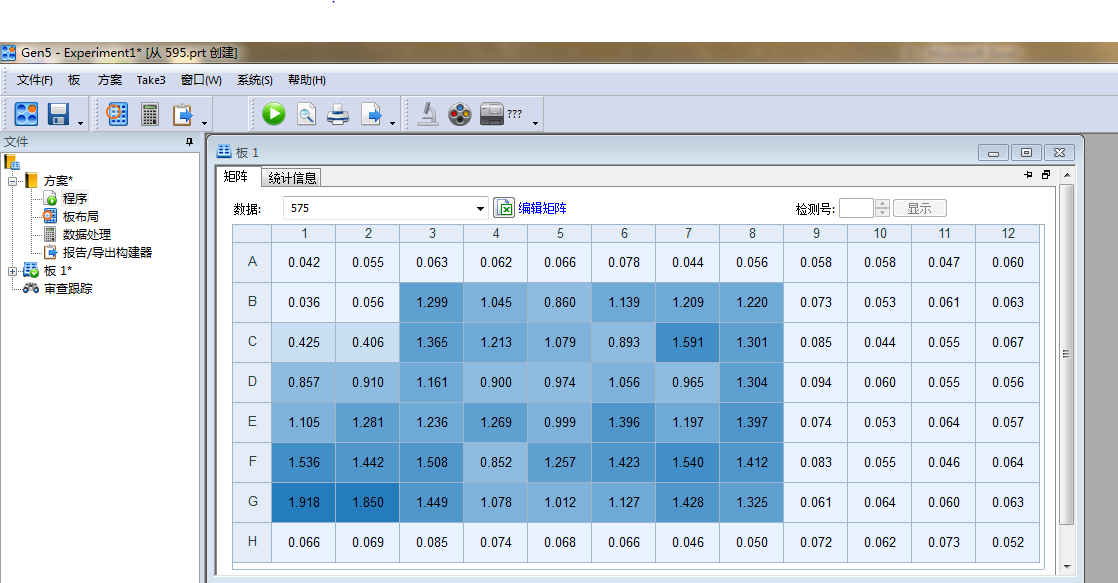

Supplement: Supplementary file 1 [file pharmaceuticals-19-00665-s001.zip › Data--Please check if this file does not need to be published/raw data/serum-ca/serum-Ca-2.PNG]

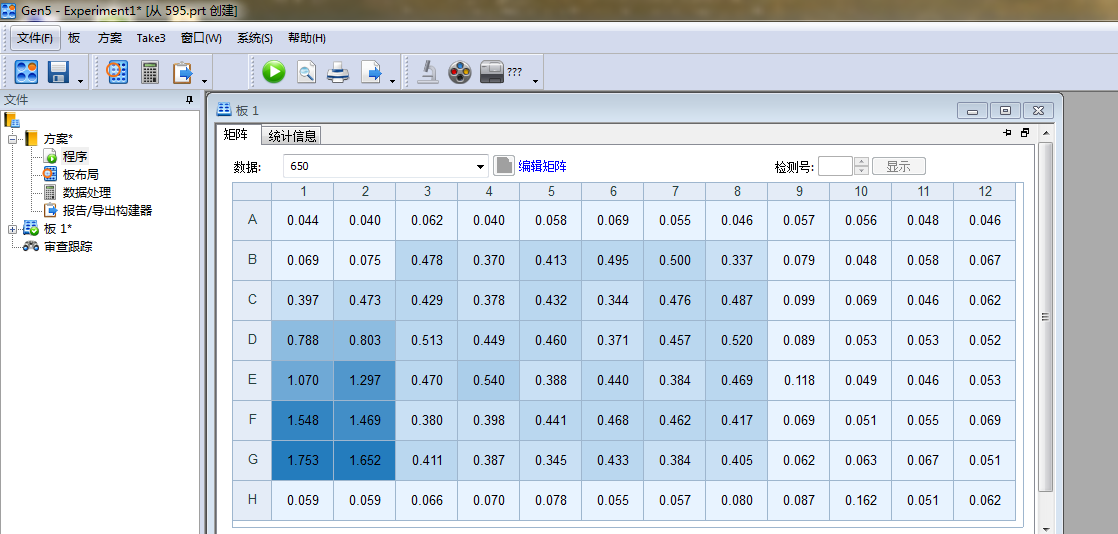

Supplement: Supplementary file 1 [file pharmaceuticals-19-00665-s001.zip › Data--Please check if this file does not need to be published/raw data/serum-p/serum-p-1.PNG]

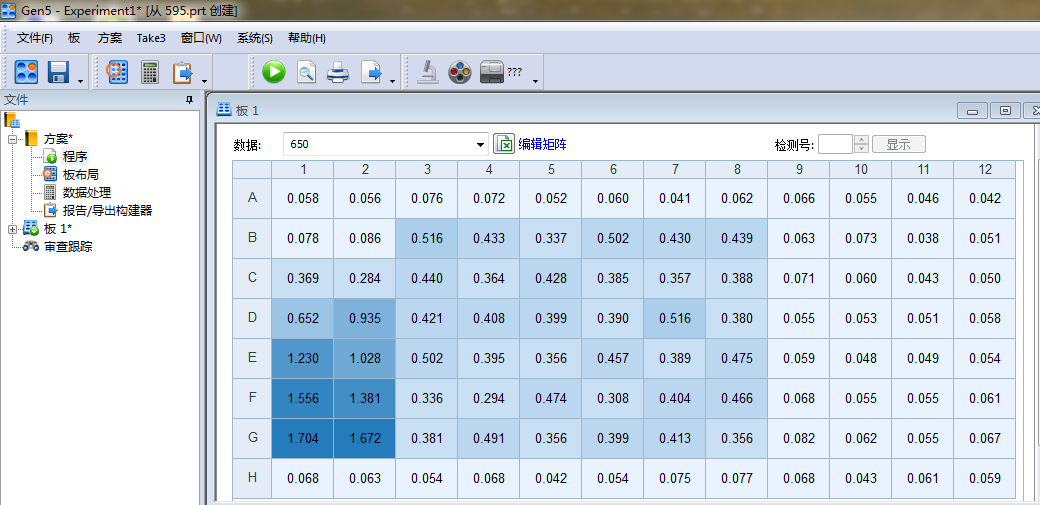

Supplement: Supplementary file 1 [file pharmaceuticals-19-00665-s001.zip › Data--Please check if this file does not need to be published/raw data/serum-p/serum-p-2.PNG]

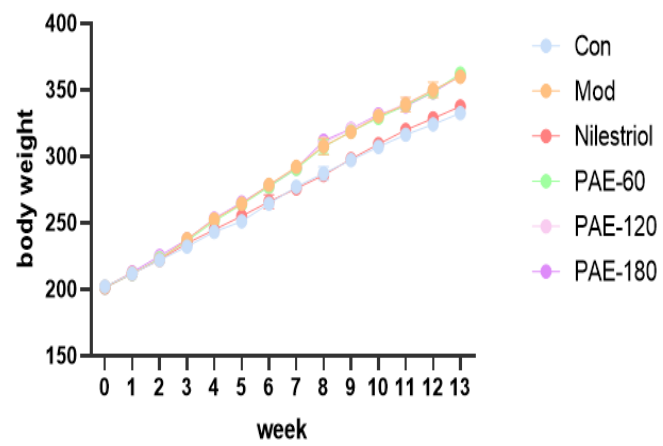

Supplementary Figure S1

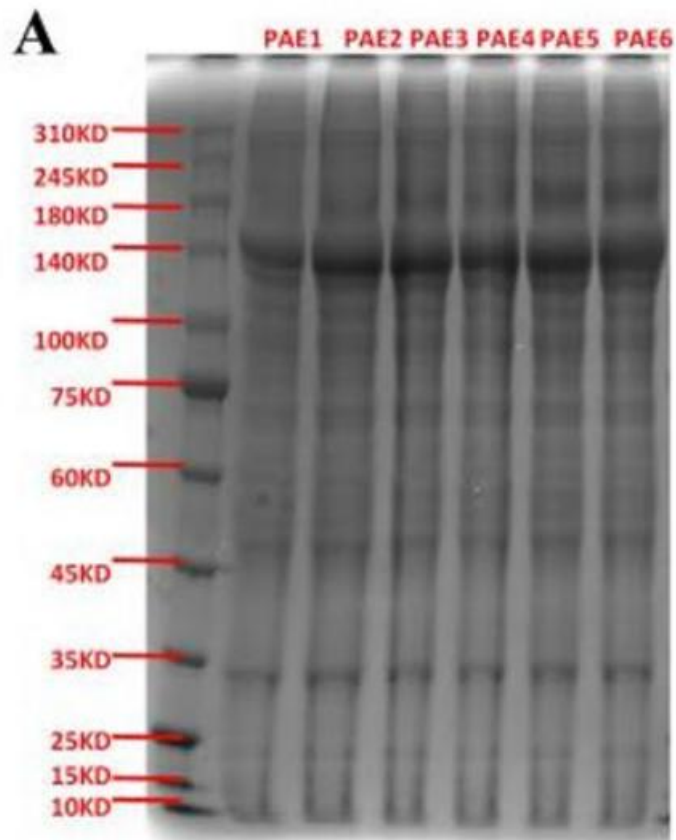

Supplementary Figure S2

Supplement: Supplementary file 1 [file pharmaceuticals-19-00665-s001.zip › Supplementary Material-Please check whether only this file need to be published/Supplementary Figure S1-S2.pdf]

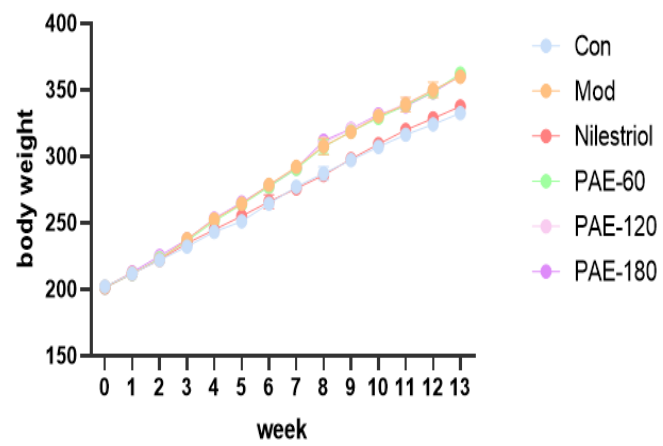

Supplementary Figure S1

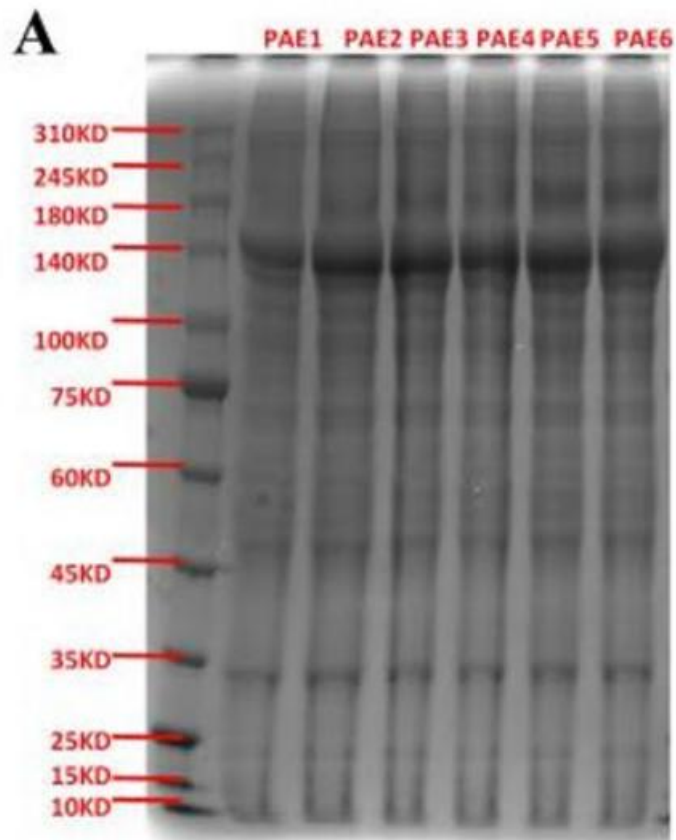

Supplementary Figure S2

Supplement: Supplementary file 1 [file pharmaceuticals-19-00665-s001.zip › Supplementary Figure S1-S2.pdf]
